# Opposing diversity–stability relationships within versus between aquatic producers and consumers

**DOI:** 10.1093/nsr/nwag274

**Published:** 2026-05-12

**Authors:** Libin Zhou, Qi Yang, Pubin Hong, Maowei Liang, Lin Jiang, Erik Jeppesen, Martin Søndergaard, Liselotte S Johansson, Qinghua Zhao, Peng Xing, Qinglong Wu, Shaopeng Wang

**Affiliations:** State Key Laboratory of Lake and Watershed Science for Water Security, Nanjing Institute of Geography and Limnology, Chinese Academy of Sciences, Nanjing 211135, China; Institute of Ecology, College of Urban and Environmental Science, and State Key Laboratory of Vegetation Structure, Function and Construction (VegLab), Peking University, Beijing 100871, China; Institute of Ecology, College of Urban and Environmental Science, and State Key Laboratory of Vegetation Structure, Function and Construction (VegLab), Peking University, Beijing 100871, China; Cedar Creek Ecosystem Science Reserve, University of Minnesota, East Bethel, MN 55108, USA; School of Biological Sciences, Georgia Institute of Technology, Atlanta, GA 30332, USA; Department of Ecoscience and Center for Water Technology (WATEC), Aarhus University, Aarhus 8000, Denmark; Sino-Danish Centre for Education and Research (SDC), Beijing 100101, China; Institute for Ecological Research and Pollution Control of Plateau Lakes, School of Ecology and Environmental Science, Yunnan University, Kunming 650500, China; Department of Ecoscience and Center for Water Technology (WATEC), Aarhus University, Aarhus 8000, Denmark; Department of Ecoscience and Center for Water Technology (WATEC), Aarhus University, Aarhus 8000, Denmark; Department of Biological Sciences, University of Notre Dame, South Bend, IN 46556, USA; State Key Laboratory of Lake and Watershed Science for Water Security, Nanjing Institute of Geography and Limnology, Chinese Academy of Sciences, Nanjing 211135, China; State Key Laboratory of Lake and Watershed Science for Water Security, Nanjing Institute of Geography and Limnology, Chinese Academy of Sciences, Nanjing 211135, China; Sino-Danish Centre for Education and Research (SDC), Beijing 100101, China; Lake Fuxian Ecological Research Station, Chinese Academy of Sciences, Yuxi 652500, China; Lake Fuxian Ecological Observation and Research Station of Yunnan Province, Yuxi 652500, China; Institute of Ecology, College of Urban and Environmental Science, and State Key Laboratory of Vegetation Structure, Function and Construction (VegLab), Peking University, Beijing 100871, China

**Keywords:** community stability, biodiversity, aquatic food webs, trophic interaction, plankton

## Abstract

While the role of biodiversity in enhancing temporal stability is well established within single trophic levels, how biodiversity at one trophic level affects stability at adjacent trophic levels remains poorly understood. To address this knowledge gap, we analyzed the relationships between diversity and stability both within and across producer (algae) and consumer (invertebrate) communities using time series from 97 aquatic food webs across the world. Within consumer communities, we found that greater species diversity was associated with increases in both population asynchrony and average population stability, leading to higher community stability. Within producer communities, producer diversity was positively associated with population asynchrony but negatively associated with population stability, resulting in no net effect. In contrast, we found consistently negative diversity–stability relationships across trophic levels: increased producer diversity was linked to decreased consumer community stability and increased consumer diversity was associated with decreased producer community stability. These negative relationships stem from adverse impacts of diversity on population stability across trophic levels, which may be due to the altered producer dynamics and intensified top-down pressure. Our findings demonstrate that incorporating antagonistic interactions between trophic levels in natural communities may alter the positive diversity–stability relationships that are typically observed in single trophic communities.

## INTRODUCTION

**Figure 1.**
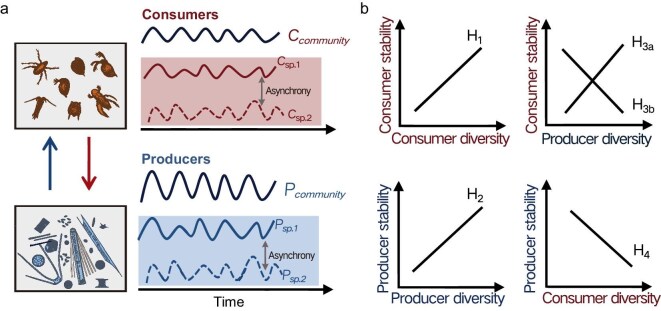
Diagram depicting diversity–stability relationships within and across trophic levels. (a) Community stability is calculated as the mean of total community abundance to its standard deviation, which is jointly determined by average stability across all populations (average population stability) and asynchrony among them (population asynchrony). In multitrophic systems, antagonistic interactions between producer (blue) and consumer (brown) communities may lead to different relationships between diversity and ecosystem stability within and across trophic levels. (b) Our hypotheses on the effects of diversity on community stability were that within trophic levels, increased diversity could contribute to stabilizing communities for both consumers (**H_1_**) and producers (**H_2_**); across trophic levels, increased producer diversity could either increase (**H_3a_**) or reduce consumer community stability (**H_3__b_**); and increased consumer diversity could reduce the producer community stability (**H_4_**). Phytoplankton image credit: Uwe Kils, CC BY-SA 3.0, via Wikimedia Commons.

Global environmental change is driving unprecedented biodiversity loss, threatening ecosystem functions and the essential services they provide to human beings [1]. Understanding how the stability of ecosystem functions will be affected by increasing environmental pressures has become one of the central challenges in ecology. While stability can be defined in various ways, temporal stability or invariability is most commonly used in empirical studies and is increasingly examined in theoretical work [2]. Temporal stability, typically measured by the amplitude of community fluctuations over time, differs fundamentally from the traditional notion of local stability that captures the return rate of a system to equilibrium following a pulse perturbation [3–5]. Mounting evidence from theoretical and experimental studies shows that biodiversity tends to promote the temporal stability of ecosystems [6–8]. These positive diversity–stability relationships emerge from increased asynchrony in populations’ responses to environmental fluctuations (population asynchrony), and/or enhanced average population-level stability due to dominance of more stable populations (average population stability) [9] (Fig. 1a). Despite these progresses, current knowledge of the diversity–stability relationship predominantly comes from studies within a single trophic level [10], especially terrestrial plants in grasslands [11] or forests [12]. However, natural ecosystems are shaped by complex interactions and feedbacks across trophic levels, which can substantially influence species dynamics and complicate the relationships between biodiversity and stability [8,10].

In multitrophic systems, community stability, i.e. the temporal stability of total community biomass or abundance at a given trophic level, can be jointly regulated by biodiversity within and across trophic levels. While theoretical models of consumer–resource interactions predict that species diversity within a trophic level typically stabilizes community biomass at that level, it could either increase or decrease the community stability at adjacent levels [13,14]. For example, producer diversity may promote consumer community stability by providing more balanced food [15]; however, when carrying capacity is constrained, increased producer diversity may decrease consumer biomass and stability by decreasing per-species producer biomass [16]—thereby limiting food for specialist consumers [15]—or by increasing the probability of including less palatable or better defended species [15,17]. Similarly, while a greater diversity of specialist consumers can stabilize their food resources by reducing the per-species consumption pressure [13], the diversity of generalist consumers may have opposite effects: increased diversity of generalist consumer could amplify the consumption pressure and reduce producer biomass [18], resulting in lower temporal stability of producer communities [13,14] and the entire food webs [19]. As a result, complex biodiversity–stability relationships across trophic levels could emerge from trophic feedbacks involving bottom-up and top-down processes, such as dietary complementarity and chemical defense of producers [20], as well as trade-offs between consumer dietary breadth and consumption rates [13].

In spite of these theoretical insights, empirical evidence on diversity–stability relationships across trophic levels remains sparse and contentious. Studies showed that plant diversity can have either positive [21] or neutral effects [22] on consumer community stability, while consumer diversity may have negative [23] or neutral [24] effects on producer community stability. The overall stability of multitrophic communities depends on the stability of constituent trophic groups [10], with higher trophic levels often exhibiting greater stability than lower ones [25]. Moreover, predators at higher trophic levels can further promote multitrophic community stability through cascading effects on lower trophic levels [23]. These empirical patterns may be explained by species diversity mediating community stability differently across trophic levels [10]. However, critical knowledge gaps persist. First, experimental studies typically examine bottom-up or top-down effects in isolation, missing the reciprocal feedbacks that govern stability in natural multitrophic communities. Second, mechanisms linking diversity at one trophic level to stability at another remain unclear. For example, it has been reported that higher algal diversity synchronizes herbivore populations in tropical marine systems, yet herbivore community stability paradoxically increases [10]. This highlights the need to explore alternative pathways beyond population synchrony, particularly average population stability [13,14]—a core theoretical mechanism that remains empirically underexplored in cross-trophic contexts.

In this study, we compiled a comprehensive dataset of time series from 97 aquatic food webs across the world in order to investigate the effects of species diversity within and across trophic levels on community stability (Fig. S1 and Table S1). We specifically examined datasets on invertebrate consumers (in total 941 genera) and algal producers (626 genera) sampled over 3–43 years across freshwater and marine ecosystems (Figs S2 and S3). We note that our analyses focus on broad producer and consumer trophic levels, with finer-scale aspects of consumer diversity (such as omnivory or trophic position) not explicitly resolved in this study. For both producer and consumer communities, we quantified community diversity by the inverse of the Simpson index and community stability by the temporal invariability of total community abundances (Fig. 1a). Here, we used an abundance-weighted diversity measure, because abundant species contribute more to community stability than rare ones [26], but we also tested the robustness of our results using Shannon diversity index and richness. In addition, extrinsic environmental factors such as temperature can influence community stability both directly, through species’ physiological responses [27], and indirectly, by modulating diversity–stability relationships [28]. To account for these effects, we included two temperature-related covariates in our analysis: annual mean temperature and its temporal variation (quantified by the standard deviation). This allows us to account for the effects of temperature and to better isolate the effects of biodiversity on temporal stability.

We developed four hypotheses regarding diversity–stability relationships within and across trophic levels based on previous evidence. Within trophic levels, we expected that consumer diversity could be positively associated with consumer stability (**H_1_**) and that producer diversity could be positively associated with producer stability (**H_2_**). However, across trophic levels, we predicted that antagonistic trophic interactions might fundamentally alter these relationships. Specifically, we hypothesized that producer diversity could either stabilize consumer communities by providing more balanced food (**H_3__a_**) or destabilize them (**H_3__b_**) due to enhanced resistance to herbivory [15] or reduce food availability [13]. Conversely, given that aquatic invertebrate consumers are predominantly generalists with high resource utilization efficiency [29], higher consumer diversity may intensify top-down pressure through complementary resource exploitation [15], leading to decreased producer community stability (**H_4_**; Fig. 1b). To elucidate the mechanisms through which diversity influences community stability both within and across trophic levels, we examined how diversity influenced population asynchrony and population stability, which together determine community stability [9] (Fig. 1a). Our study advances the understanding of diversity–stability relationships in multitrophic ecosystems, building on and extending the classic notion derived primarily from single trophic systems.

## RESULTS

**Figure 2.**
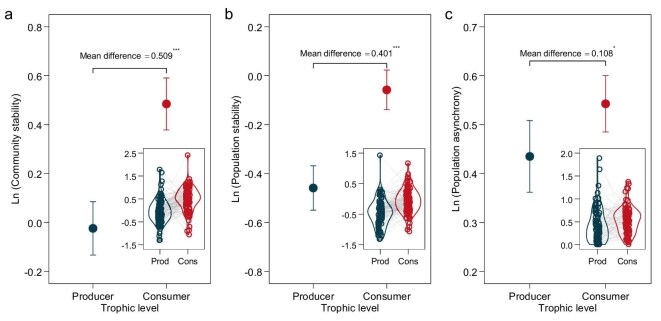
Paired comparisons between producer and consumer communities: (a) community stability, (b) population stability, and (c) population asynchrony. The paired open dots represent independent sampling sites in our datasets. Note that the stability metric data were ln-transformed. Colored symbols represent mean ± 2 standard errors. The numbers represent the log-scale mean difference (i.e. log-ratio) in the respective metric between producer and consumer communities. Statistics from paired *t*-tests are indicated as follows: ***P < 0.001, *P < 0.05.

We found that consumer communities had greater temporal stability compared to producer communities (log-ratio = 0.509, *P* < 0.001; Fig. 2a). This was attributed mainly to higher average population stability in the consumer communities (log-ratio = 0.401, *P* < 0.001; Fig. 2b), even though population asynchrony was also higher for consumers (log-ratio = 0.108, *P* = 0.017; Fig. 2c).

**Figure 3.**
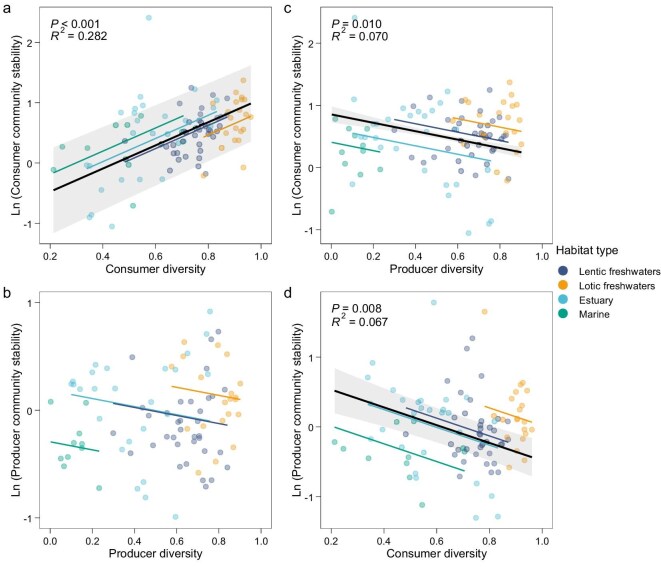
Relationships between biodiversity and community stability within and across trophic levels: (a) consumer diversity versus consumer community stability, (b) producer diversity versus producer community stability, (c) producer diversity versus consumer community stability, and (d) consumer diversity versus producer community stability. Colored lines represent the best-fit trendline in the linear models within each habitat type. Black solid lines indicate statistically significant relationships derived from LMMs with random intercepts, with shaded areas representing 95% confidence intervals.

Within trophic levels, we found higher consumer stability at sites with higher consumer diversity (supporting hypothesis **H_1_**; F_1,32.8_ = 36.76, *P* < 0.001; Fig. 3a and Tables S2 and S3), whereas there was no relationship between producer diversity and producer community stability (rejecting hypothesis **H_2_**; F_1,70.5_ = 1.09, *P* = 0.300; Fig. 3b and Table S2). In contrast, we found lower consumer stability with higher producer diversity (supporting hypothesis **H_3__b_** but not **H_3a_**; F_1,53.5_ = 7.07, *P* = 0.010; Fig. 3c and Table S2) and lower producer stability with higher consumer diversity (supporting hypothesis **H_4_**; F_1,70_ = 7.54, *P* = 0.008; Fig. 3d and Tables S2 and S3). We found no influence of mean annual temperature on the community stability of consumers (F_1,26.6_ = 0.36, *P* = 0.555) or producers (F_1,91.2_ = 0.64, *P* = 0.427). However, producer community stability was marginally negatively related to the temporal variation of temperature (F_1,89.9_ = 3.126, *P* = 0.080; Table S2), while there was no relationship with consumer community stability (F_1,71.4_ = 0.75, *P* = 0.389; Table S2).

These findings remained robust across comprehensive sensitivity analyses. The observed diversity–stability relationships across trophic levels persisted when analyses were restricted to longer time series (≥5 years; Fig. S4 and Table S4) and when habitat type and its interactions with diversity measures were incorporated as covariates (Table S5). Results remained qualitatively unchanged using alternative diversity metrics: Shannon diversity and taxonomic richness after excluding extremely rare genera (e.g. those with average population size smaller than 1‰ [30]; Figs S5–S7 and Table S4), although patterns associated with producer diversity varied if the original taxonomic richness (including extremely rare genera) was used (Table S4). The robustness of our results was further confirmed when accounting for the seasonality in sampling intensity (Table S6), and when spatial autocorrelation was explicitly controlled using generalized additive mixed-effects models (GAMMs; Table S7).

**Figure 4.**
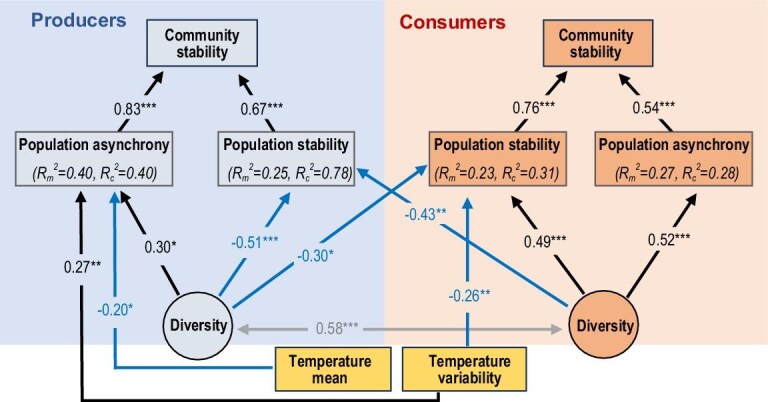
SEM illustrating the relationships among diversity, stability metrics, and temperature variation across the two trophic levels. The diagram presents the final SEM with statistically significant pathways (*P* < 0.05) and their corresponding standardized path coefficients. Positive associations are indicated by black arrows and negative relationships by blue arrows. Gray arrows represent bivariate correlations. The proportions of variance explained by the linear mixed models are denoted by marginal R² (R_m_²), which represents the variance explained by the fixed effects only, and conditional R² (R_c_²), which accounts for both fixed and random effects. All stability indices were ln-transformed prior to analysis. The final model demonstrated good fit with Fisher’s C = 33.55, degrees of freedom (d.f.) = 40, and *P* = 0.75. Full SEM is provided as Fig. S8.

Our structural equation model (SEM) revealed distinct pathways through which diversity influenced community stability within versus across trophic levels (Fig. 4). Within trophic levels, consumer diversity was positively associated with both consumer population stability and consumer population asynchrony, which together led to greater consumer community stability. In contrast, producer diversity was associated with higher producer population asynchrony but decreased producer population stability. These opposing effects counterbalanced each other, such that there was no net effect on producer community stability. Across trophic levels, consumer diversity was negatively associated with producer population stability, resulting in decreased producer community stability. Similarly, producer diversity was negatively associated with consumer population stability, resulting in decreased consumer community stability. Temperature was differentially associated with the different components of stability. Mean annual temperature was negatively associated with producer population asynchrony, while the temporal temperature variability had contrasting relationships with the stability of producer and consumer communities. Specifically, producer population asynchrony increased, while the population stability of consumers decreased, as the temperature variability increased (Fig. 4).

## DISCUSSION

Diversity–stability theory has been well established through decades of studies in trophically simplified ecosystems, particularly terrestrial plants [7,11], but its applicability to complex multitrophic systems remains elusive [10]. By analyzing 97 aquatic food webs from sites primarily in North America and Europe, we synthesized the relationships within and across trophic levels in natural consumer–producer systems and revealed patterns that both support and refine established paradigms. We found that aquatic invertebrate consumers were more stable than algal producers [25], predominantly due to their higher population stability. Our analyses showed that consumer diversity promoted the stability of consumer communities, providing additional evidence from heterotrophs for the positive diversity–stability relationships [31], whereas no such relationships were found within producer communities. In contrast, we found that biodiversity at one trophic level had consistently adverse impacts on the community stability at adjacent trophic levels.

The difference in diversity–stability relationships between producers and consumers is attributed to different mechanisms underlying stability at the two trophic levels. Consumer diversity enhanced both population asynchrony and population stability, collectively contributing to increased consumer community stability, whereas producer diversity promoted population asynchrony but suppressed population stability, resulting in no net effect on producer community stability. In other words, producer and consumer diversity provide consistent insurance effects through increased population asynchrony [32,33], but their opposite effects on population stability led to contrasting effects on community stability within respective trophic levels. The destabilizing effects of producer diversity on producer populations may be understood from the strong competition for limited resources among aquatic producers [3,8]. In contrast, aquatic consumers have higher movement ability [34] and broader dietary spectrum [29], enabling populations to adopt flexible feeding strategies in diverse communities [35], which helps buffer their populations against environmental fluctuations [36].

The consistently destabilizing effects of diversity on adjacent trophic levels may be attributed to several mechanisms associated with antagonistic interactions across trophic levels. From a bottom-up perspective, higher diversity of producers may alter their overall availability to consumers through various pathways: enhanced resistance, where less palatable species protect vulnerable ones [37]; disrupted foraging patterns, as frequent prey switching prevents the development of optimal foraging strategies [38]; and increased foraging costs, manifested in longer search times and higher handling costs for consumers [39]. From a top-down perspective, increased consumer diversity may enhance the magnitude of predation pressure through complementary exploitation [15], amplifying temporal fluctuations in producer populations. In addition, increased population asynchrony of consumers may result in more unpredictable patterns of resource exploitation [40]. In all, antagonistic trophic interactions can disrupt the stabilizing effects of diversity typically observed within single trophic levels [13].

Within trophic levels, previous studies have shown that both the mean state and temporal variability of climate can influence ecosystem stability [27]. By simultaneously examining relationships between climate and population dynamics across multiple trophic levels, we advance our understanding in this area. Algal producers are characterized by high turnover rates [41], which allow them to quickly respond to environmental variation. Our observed decrease in producer population asynchrony with higher temperatures suggests its high vulnerability to perturbations. However, higher population asynchrony in response to high temperature variability complicates the effects of climate change on aquatic ecosystems, especially under frequent extremes [42]. While mean temperature had limited effects on the stability of invertebrate consumers, the temporal variability in temperature was a critical driver in reducing consumer population stability. But this was not the case for producers. This suggests a potential decoupling of producer–consumer dynamics [43], which may subsequently impair food web functions such as energy transfers and trophic cascades [44]. This implies that future climate scenarios with increased temperature variability might lead to more unstable aquatic communities, particularly within higher trophic levels.

While theory on the relationship between diversity and stability has made substantial progress [3,19,45], a significant challenge remains in transporting these principles to natural communities where species interactions engage in complex networks. Our research addresses this gap by elucidating diversity–stability relationships through the lens of population dynamics in multitrophic communities. Classic theory has demonstrated that antagonistic consumer–resource interactions can induce instability in the form of population oscillations that propagate through food webs [46]. In line with this theory, our results suggest that while diversity effects may stabilize communities within a single trophic level, it may cause instability and amplify the temporal variability in populations at adjacent trophic levels. To further advance these findings, future studies could expand the current bitrophic framework by incorporating higher-level predators such as piscivorous fish, the presence of which can fundamentally reshape interaction networks. Recent theoretical work predicts that aquatic consumers—which exhibit higher metabolic rates than their unicellular resources—can impose stronger top-down effects and thereby generate cascading stability patterns, i.e. nonmonotonic changes of stability with increasing trophic levels [47]. In addition, integrating nutrient dynamics would provide crucial insights into how anthropogenic eutrophication modulates diversity–stability relationships across the entire food webs in natural systems.

Our findings have important implications for conservation strategies in an era of unprecedented biodiversity loss. While traditional efforts often aim to maximize biodiversity indiscriminately, our results suggest that trophic-specific strategies should be tailored to the desired ecosystem service goals. For example, in aquatic ecosystems where water quality is the primary concern, conserving producer diversity may enhance resource use efficiency and water purification services [48], though potentially at the cost of destabilizing consumer populations. Conversely, when the primary goals are fish production or nutrient cycling, prioritizing consumer diversity could be more effective. Furthermore, our results underscore that restoration efforts, such as predator reintroductions, should account for both their direct effects on community composition and indirect consequences for stability across trophic levels [47]. By highlighting the key role of dominant species in driving multitrophic stability, our study also challenges the sufficiency of species richness alone as a conservation target. Instead, maintaining appropriate abundance distributions and functional roles across trophic levels is equally critical. Ultimately, effective conservation in the Anthropocene requires a nuanced, multitrophic perspective that balances biodiversity conservation within and across trophic levels to safeguard both ecosystem stability and functioning under increasing environmental variability [49].

## METHODS

### Data compilation

To investigate biodiversity effects on community stability within and across trophic levels, we compiled temporal observational data of aquatic ecosystems where population-level temporal records across trophic levels are most available. Data were filtered according to the following criteria: (i) concurrent sampling of algal producers and invertebrate primary consumers, (ii) records with minimum of 3 years for both trophic levels, and (iii) taxa identified to at least genus level. The above criteria resulted in 97 sites spanning four habitat types: lentic freshwater (lakes, *n* = 37), lotic freshwater (streams, *n* = 21), estuaries (*n* = 28), and marine ecosystems (*n* = 11), with monitoring durations of 3–43 years (Figs S1–S3 and Table S1). To standardize taxonomic resolution, we aggregated species-level data to genus level by summing the abundance values of all species belonging to the same genus, yielding 1567 genera (626 algal and 941 consumer genera). To control for within-year seasonal fluctuations of each site, we first converted raw records into seasonal-level data, then filtered records to ensure consistent seasonal coverage between trophic levels and across years, and finally averaged across seasons to derive annual abundance values.

We also extracted site-specific air temperature from the CRU TS v4.07 [50] and NOAA ERSST v5 [51] datasets for freshwater and marine sites, respectively, filtered to match biological sampling periods. Air temperature was used as a proxy for water temperature given their strong correlation in aquatic ecosystems [52].

### Biodiversity and stability metrics

Community stability was defined as the inverse of the coefficient of variation (1/CV) of total community abundance [7], and decomposed into population stability and population asynchrony [9]:


\begin{eqnarray*}
{\mathrm{Community\ stability}} = \frac{{\sum}{\mu }_i}{{\sqrt {\sum {v}_{i,j}} }},
\end{eqnarray*}

\begin{eqnarray*}
{\mathrm{Population\ stability}} = \frac{{\sum {\mu }_i}}{{\sum \sqrt {{v}_{ii}} }},
\end{eqnarray*}

\begin{eqnarray*}
{\mathrm{Population\ asynchrony}} = \frac{{\sum \sqrt {{v}_{ii}} }}{{\sqrt {\sum {v}_{i,j}} }},
\end{eqnarray*}

where ${\mu }_i$ is the temporal mean of population *i*, and ${v}_{i,j}$ is the temporal covariance between populations *i* and *j*. All three stability indices were calculated for both trophic levels at each site. Biodiversity was quantified as the Simpson diversity index given it provides more accurate predictions of diversity effects on stability compared to the richness-based index [53].

### Statistical analysis

To compare stability metrics between producers and consumers, we used paired *t*-tests on the ln-transformed data, with observations paired by sites to account for within-site comparisons. To examine diversity–stability relationships within and across trophic levels, we applied linear mixed-effects models (LMMs), with ln-transformed community stability as the response variable, and producer and consumer diversity, annual mean, and variability of temperature as predictor variables. Habitat type was included as a random effect on the intercept to control for habitat-specific variation. To explore mechanisms linking diversity to stability, we employed piecewise structural equation modelling [54], incorporating LMMs for paths involving population stability and asynchrony, and linear models for direct paths to community stability.

Robustness of our results was assessed through: (i) multiple linear regression models with habitat type as a fixed factor; (ii) analysis of data with minimum 5 years; (iii) alternative diversity metrics including richness and Shannon index; (iv) analysis of data with varying seasonal coverage; and (v) GAMMs accounting for spatial autocorrelation [55].

Detailed description of the Methods section is provided in the Supplementary data.

## Supplementary Material

nwag274_Supplemental_File

## Data Availability

The dataset required to interpret, verify, and extend the research in the manuscript is available at GitHub repository (https://github.com/LibinZhoueco/Aquatic-Multi-trophic-Stability). Scripts for running analyses underlying this study’s results are also publicly available in a GitHub repository (https://github.com/LibinZhoueco/Aquatic-Multi-trophic-Stability). Any relevant raw data can be found within the article and its Supplementary data.
