# Supplementary material for: Opposing diversity–stability relationships within versus between aquatic producers and consumers
Source: Natl Sci Rev. 2026 May 12;13(11):nwag274. doi: 10.1093/nsr/nwag274 (PMC13281100; doi:10.1093/nsr/nwag274)
Supplement: nwag274_Supplemental_File [file nwag274_supplemental_file.docx]

**Supplementary data for:**

**Opposing diversity-stability relationships within versus between aquatic producers and consumers**

Libin Zhou^1^, Qi Yang^2^, Pubin Hong^2^, Maowei Liang^3^, Lin Jiang^4^, Erik Jeppesen^5,6,7^, Martin Søndergaard^5^, Liselotte S. Johansson^5^, Qinghua Zhao^8^, Peng Xing^1*^, Qinglong Wu^1,6,9,10*^, Shaopeng Wang^2*^

1. State Key Laboratory of Lake and Watershed Science for Water Security, Nanjing Institute of Geography and Limnology, Chinese Academy of Sciences, Nanjing, China
2. Institute of Ecology, Key Laboratory for Earth Surface Processes of the Ministry of Education, College of Urban and Environmental Sciences, Peking University, Beijing, China
3. Cedar Creek Ecosystem Science Reserve, University of Minnesota, East Bethel, Minnesota, USA
4. School of Biological Sciences, Georgia Institute of Technology, Atlanta, GA, USA
5. Department of Ecoscience and Center for Water Technology (WATEC), Aarhus University, 8000 Aarhus, Denmark
6. Sino-Danish Centre for Education and Research (SDC), Beijing, China.
7. Institute for Ecological Research and Pollution Control of Plateau Lakes, School of Ecology and Environmental Science, Yunnan University, Kunming, China
8. Department of Biological Sciences, University of Notre Dame, South Bend, USA
9. Lake Fuxian Ecological Research Station, Chinese Academy of Sciences, Yuxi, 652500
10. Lake Fuxian Ecological Observation and Research Station of Yunnan Province, Yuxi 652500

**Supplementary Data**

Methods

Figures S1 to S8

Tables S1 to S7

Supplementary References

**METHODS**

**Data compilation**

To investigate biodiversity effects on community stability within and across trophic levels, we compiled temporal observational data of population dynamics at different trophic levels. To this end, we scanned data of aquatic ecosystems where population-level temporal records across trophic levels are most available. Specifically, we collected and filtered data according to the following criteria: (1) concurrent sampling of both algal producers and invertebrate primary consumers, (2) a minimum of three years of data for both trophic levels, and (3) taxa identified to at least genus level. To standardize taxonomic resolution, we aggregated species-level data to genus level by summing the abundance values of all species belonging to the same genus. This resulted in a total of 1,567 genera (626 algal and 941 consumer genera) after excluding carnivorous invertebrates. We ensured that each site followed standardized sampling methods and sample processing protocols (including taxonomic identification procedures) throughout the duration of monitoring. In particular, to minimize the influence of variation in the seasonality of sampling, we implemented a multi-stage approach: First, we converted the original records into seasonal-level data, which denotes the (average) abundance within the season (Spring = March/April/May; Summer = June/July/August; Autumn = September/October/November; Winter = December/January/February). For instance, for a site at a given year, if abundance data were surveyed in March, July, and August, we derived the seasonal data for Spring by the records in March, and that for Summer by the average of records in July and August. Second, for each site, we filtered the seasonal records to ensure consistency between producers and consumers and across years, e.g., excluding seasonal records available only for producer or consumer or observed only in certain years. This controls for the potential bias induced by seasonality in the calculation of temporal stability. Third, we further converted seasonal data into annual values (i.e., average across seasons) to capture year-to-year variation while reducing the influence of within-year seasonal fluctuations. Annual data were derived by averaging the abundance of each trophic group across sampling months.

The above criterion led to a dataset consisting of 97 sites from four habitat types (Figs. S1-3): lentic freshwater (lakes, n = 37; unit: individual per liter), lotic freshwater (inland streams, n = 21; unit: individual per square meter), estuaries (estuary rivers, n = 28; unit: individual per liter), and marine ecosystems (n = 11; unit: individual per square meter). Each site includes information of population dynamics over 3 to 43 years. These data were obtained from three main sources (Table S1): (1) 65 datasets were obtained from publicly available databases, including LTER datasets publicly available from Environmental Data Initiative (n=64) and Biodiversity Time Series (n=1), (2) 21 datasets from the Danish National Monitoring Program, and (3) 11 datasets from direct requests to researchers.

We also extracted site-specific temperature data from the Climatic Research Unit Time Series dataset, version 4.07 (CRU TS v4.07) [1] and the NOAA Extended Reconstructed Sea Surface Temperature dataset, version 5 (ERSST, v5) [2], respectively for freshwater and marine ecosystems. Air temperature data were collected due to its strong correlation with water temperatures in aquatic ecosystems [3]. To ensure the direct effects of temperature on the stability of the two trophic levels, we filtered temperature data to match the biological sampling periods on a monthly basis. These data were subsequently averaged to annual values.

**Biodiversity and stability calculation**

We defined community stability as the inverse of coefficient of variation (1/CV) of total community abundance [4]. We decomposed community stability into two key components [5]: the weighted average of population stability across population and population asynchrony. These components are mathematically expressed as:

|  | Community stability = $\frac{\sum\mu_{i}}{\sqrt{\sum v_{i,j}}}$ | (1) |
| --- | --- | --- |
|  | Population stability = $\frac{\sum\mu_{i}}{\sum\sqrt{v_{ii}}}$ | (2) |
|  | Population asynchrony = $\frac{\sum\sqrt{v_{ii}}}{\sqrt{\sum v_{i,j}}}$ | (3) |

where $\mu_{i}$ represents the temporal mean of population i, and $v_{i,j}$ denotes the temporal covariance between population i and j. We calculated the three stability indices for both producers and consumers at each site. We note that these stability metrics are unit-free, so the difference in unit (individual per liter, or individual per square meter) did not introduce analytical bias or affect the comparability across habitat types. Given that previous findings have shown that abundant species contributed more to community stability [5,6] and the Simpson-based diversity index provided more accurate predictions of diversity effects on stability compared to the richness-based index [7], we calculated the Simpson diversity index for each trophic group at each time step, then averaged these values over time to obtain a mean diversity value for each site. In addition, we estimated the temporal variation of temperature for each site as the standard deviation across years.

**Statistical analysis**

To examine the relationships between diversity and stability across trophic levels, we conducted two sets of analyses. First, we compared stability metrics between producers and consumers using paired t-tests on the ln-transformed data, with observations paired by sites to account for within-system comparisons (Fig. 2). To investigate how biodiversity affects community stability both within and across trophic levels, we then developed linear mixed-effects models with ln-transformed community stability of either consumers (for **H_1_** and **H_3_**) or producers (for **H_2_** and **H_4_**) as response variables. Producer and consumer diversity served as key predictor variables, while annual mean temperature, and annual variation of temperature were included as additional fixed effect covariates to account for potential confounding effects. Habitat type was included as a random effect on the intercept to control for habitat-specific variation. Notably, given the strong confounding between sampling protocols and habitat types in our dataset (for example sampling protocols were identical for marine ecosystems), the random effects represent combined habitat-protocol influences rather than habitat type alone. Specifically, to examine how both consumer diversity (**H_1_**) and producer diversity (**H_3_**) influence consumer stability, we analyzed their effects on ln-transformed consumer community stability. Similarly, to test how producer diversity (**H_2_**) and consumer diversity (**H_4_**) affect producer stability, we explored their effects on ln-transformed producer community stability. In both analyses, we accounted for environmental covariates by including annual mean and variations of temperature. To assess the proportion of variance explained by single fixed factors, we calculated the marginal R² values for models containing significant fixed effects using the r2beta function from the “r2glmm” R package [8]. The linear mixed models were performed using the “lmer” function from the “lme4” package [9].

To understand how diversity affects community stability through population asynchrony and population stability both within and across trophic levels, we employed a piecewise structural equation model (pSEM) using the R package “piecewiseSEM” [10]. We initially characterized the effects of producer and consumer diversity on population stability and population asynchrony at both trophic levels using linear mixed models with the “lmer” function, with producer diversity, consumer diversity, annual mean and variation of temperature as fixed factors, and habitat type as a random factor. We estimated direct paths from population stability and population asynchrony to community stability for both trophic groups using linear models with the “lm” function. Our initial models also incorporated multiple bivariate correlations between producer and consumer diversity, as well as correlations among stability indices between producers and consumers. We fitted the model and applied Shipley’s test of d-separation to identify potential missing paths. We derived the final SEM by eliminating non-significant paths and variables, thereby refining our understanding of the complex interactions between diversity and stability across trophic levels.

**Sensitivity analyses**

To test the robustness of our results, we conducted comprehensive sensitivity analyses. First, we additionally conducted multiple linear regression models taking habitat type, as well as its interactive effects with producer diversity and consumer diversity as fixed factors. Second, to address potential concerns about the minimum time series length threshold, we applied more stringent criteria: examining datasets with minimum duration of 5 years (n = 82). Third, we evaluated the consistency of diversity-stability relationships using alternative diversity metrics: Shannon diversity index and richness (both with and without rare genera with average population size < 1‰ of total abundance [11]). Fourth, recognizing potential effects of the seasonality of sampling, we re-analyzed diversity-stability relationships across sites with varying seasonal coverage: 4 seasons (n = 60), ≥ 3 seasons (n = 83), and ≥ 2 seasons (n = 90). Finally, to account for spatial autocorrelation arising from the uneven geographic distribution of study sites, we employed generalized additive mixed models (GAMMs) [12] incorporating thin plate spline smooth functions for spatial coordinates (latitude and longitude) and random intercepts for habitat type. These analyses examined whether our conclusions were robust to different filtering thresholds (Figs. S4-6). All analyses were conducted in R version 3.6.0 [13].

**Figures**


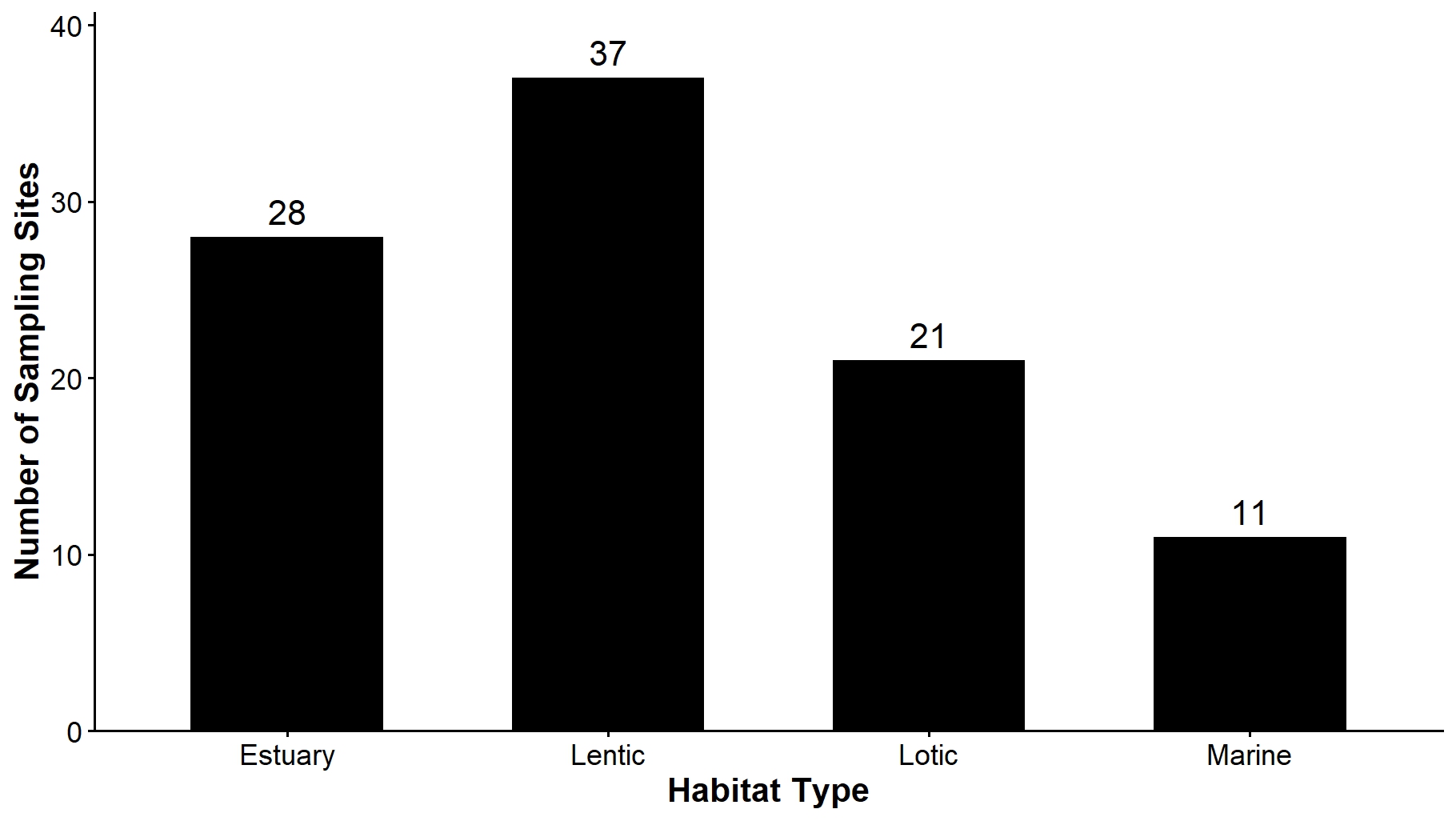


**Fig. S1** **Number of sampling sites for each habitat type.** Four habitat types were classified: estuary, lentic freshwaters (lakes), lotic freshwaters (inland streams and rivers), and marine ecosystems.


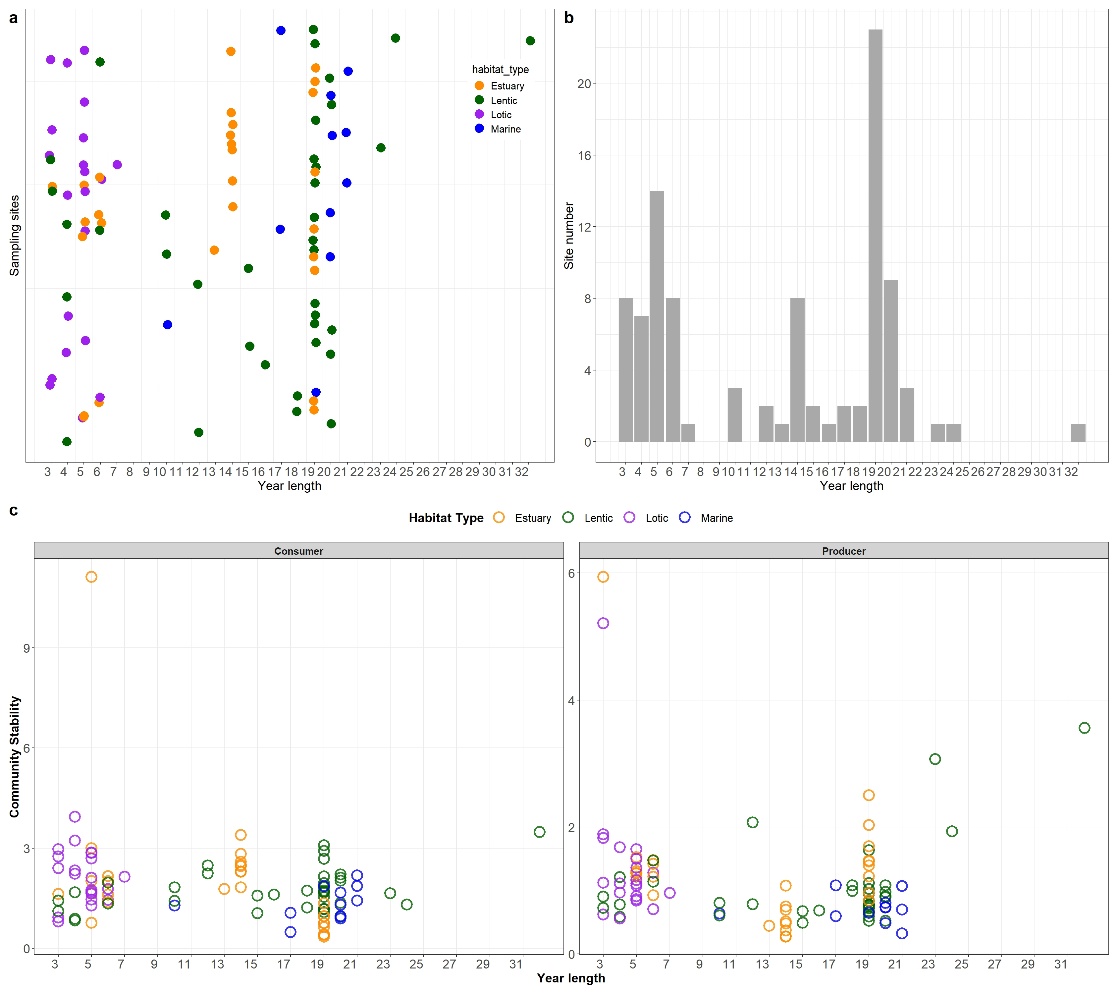


**Fig. S2** Sampling effort over time: (a) distribution of monitoring sites by years of data collection, (b) site count for each monitoring duration and (c) community stability as responses to sampling years for both functional groups.


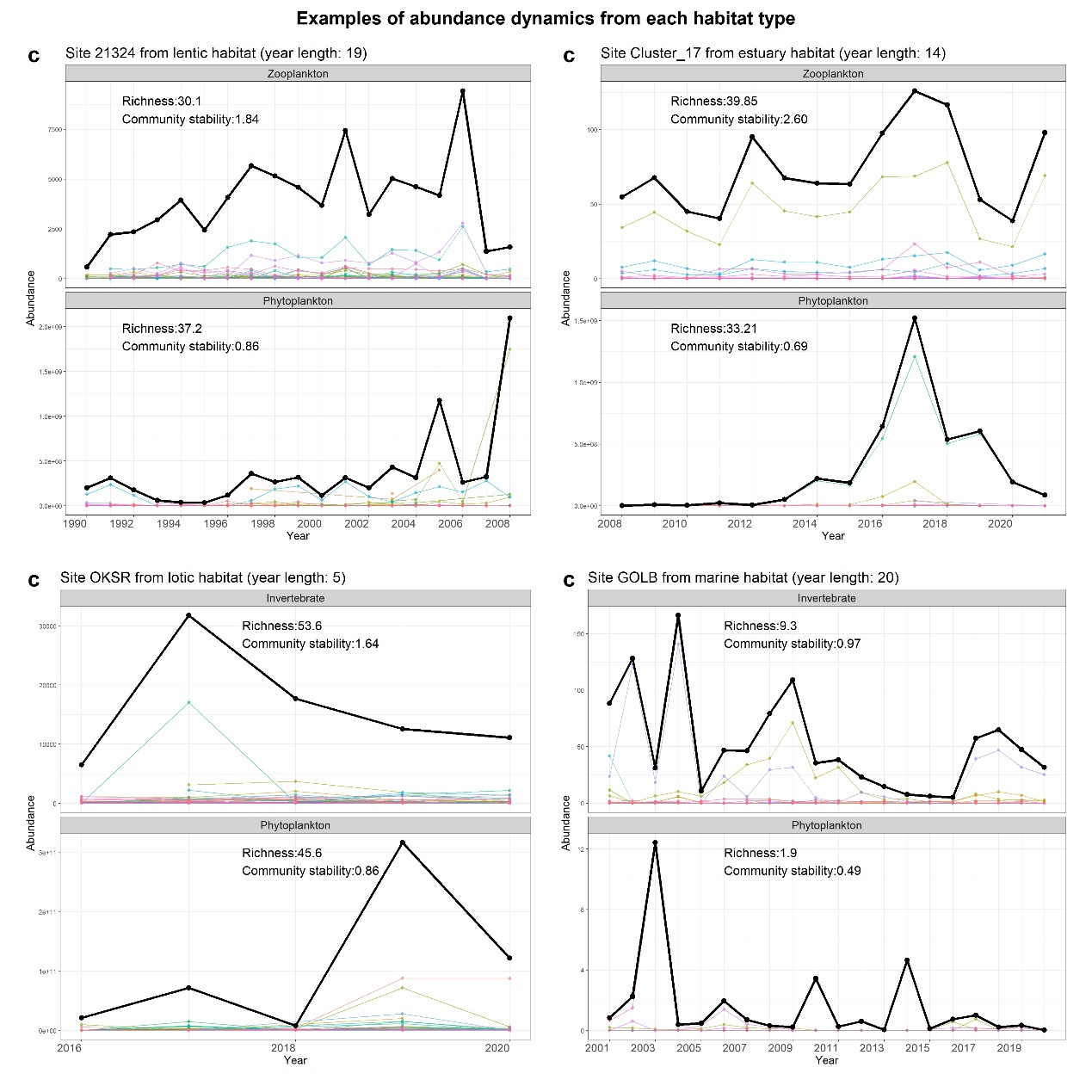


**Fig. S3** Examples of abundance dynamics from four habitat types. The sites displayed represent time series with durations equal to the median sampling period length for each respective habitat type. The bold black lines reflect the temporal dynamics of total biomass, while the colored lines reflect dynamics of each population.

**
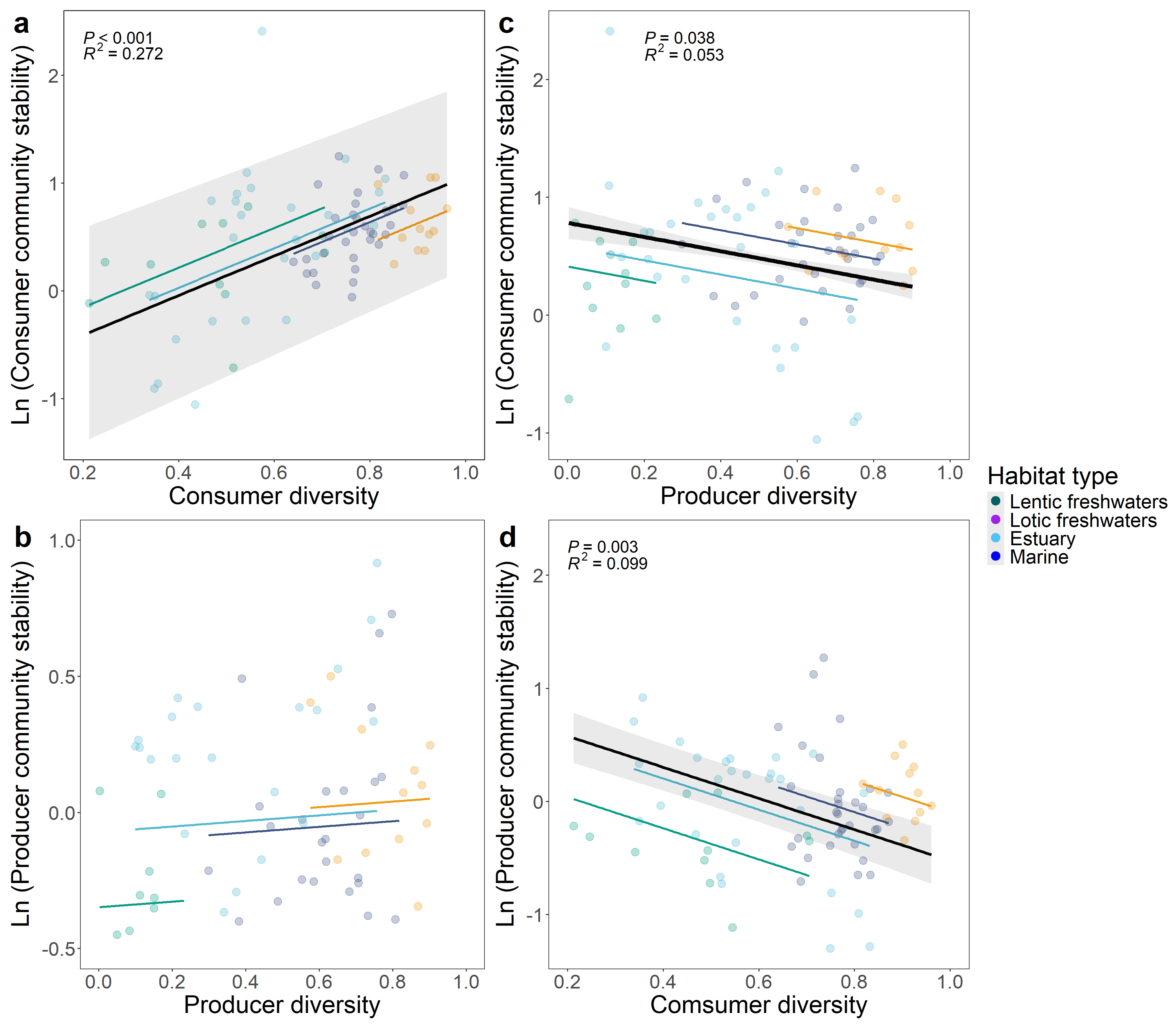
**

**Fig. S4 Relationships between Simpson diversity and community stability within and across trophic levels, derived from datasets with a minimum of 5 sampling years:** (**a**) consumer diversity vs. consumer community stability, (**b**) producer diversity vs. producer community stability, (**c**) producer diversity vs. consumer community stability, (**d**) consumer diversity vs. producer community stability. Colored lines represent relationships within each habitat type. Black solid lines indicate statistically significant relationships derived from linear mixed-effects models with random intercepts, with shaded areas representing 95% confidence intervals.


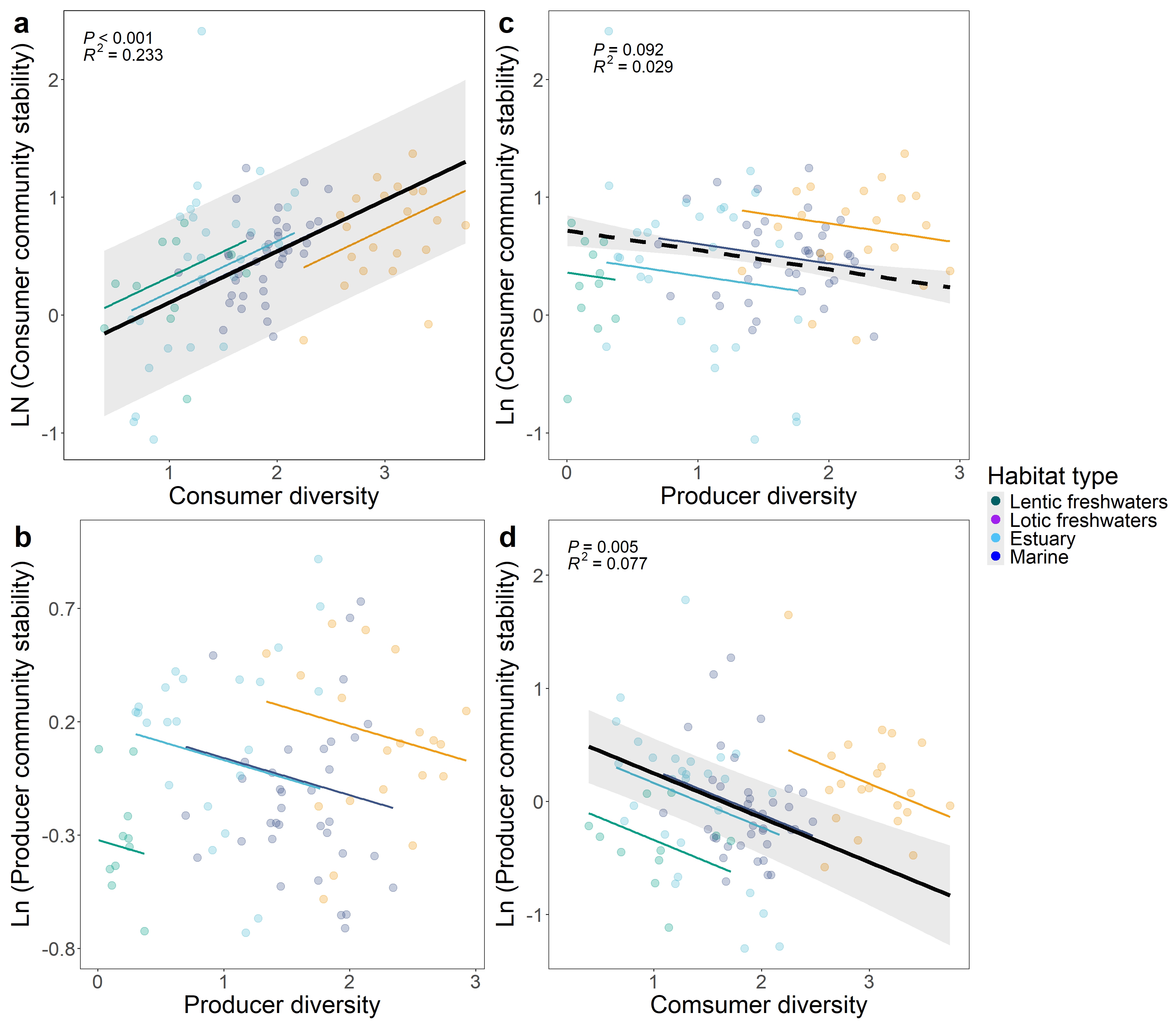


**Fig. S5 Relationships between Shannon diversity and community stability within and across trophic levels:** (**a**) consumer diversity vs. consumer community stability, (**b**) producer diversity vs. producer community stability, (**c**) producer diversity vs. consumer community stability, (**d**) consumer diversity vs. producer community stability. Colored lines represent relationships within each habitat type. Black solid lines indicate statistically significant relationships derived from linear mixed-effects models with random intercepts, and the dashed line represents a marginally significant (P<0.1) relationship. The shaded areas represent 95% confidence intervals.

**
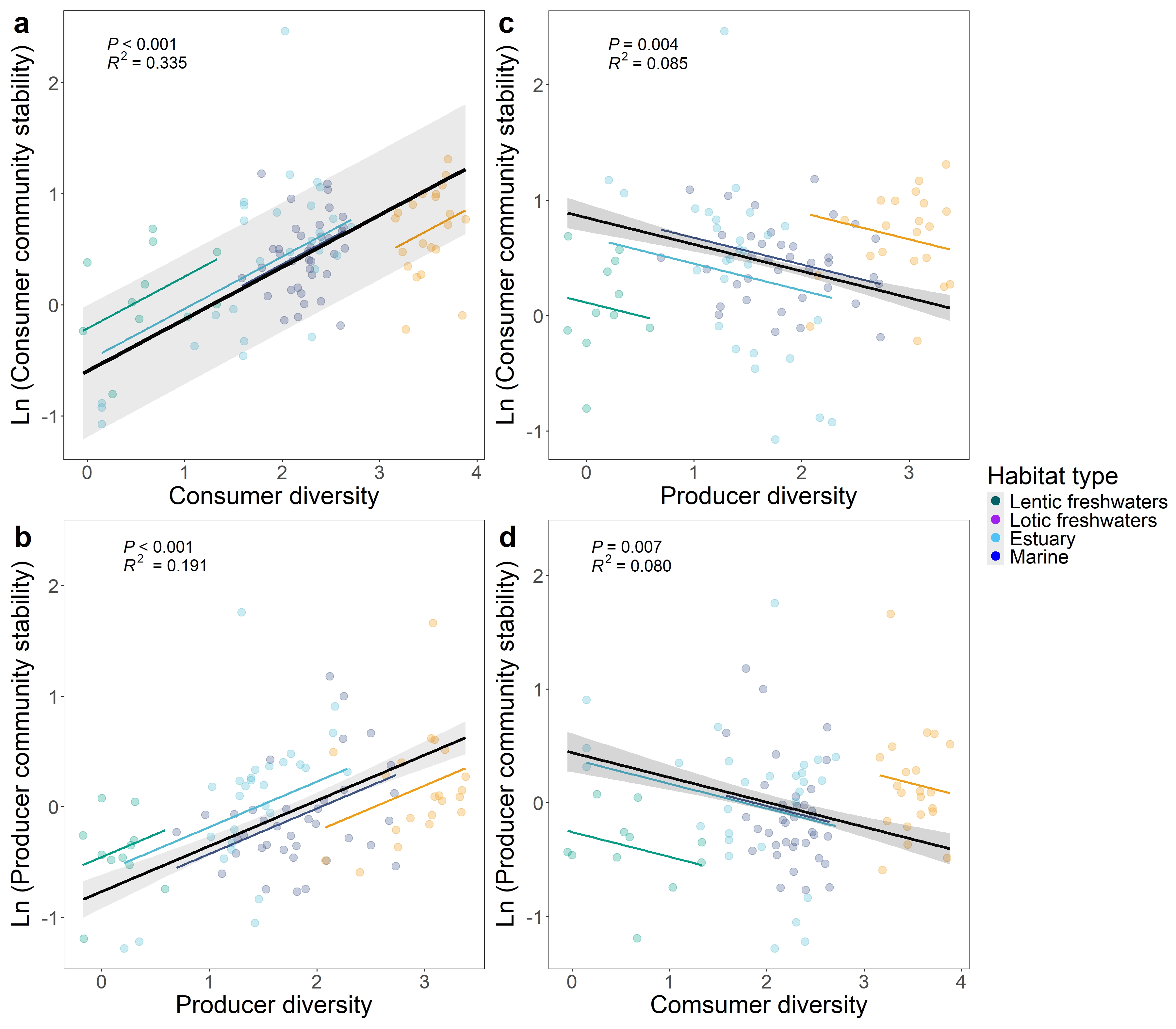
**

**Fig. S6 Relationships between Richness and community stability within and across trophic levels, with removal of rare genera (average abundance less than 1‰)**: (a) consumer diversity vs. consumer community stability, (b) producer diversity vs. producer community stability, (c) producer diversity vs. consumer community stability, (d) consumer diversity vs. producer community stability. Colored lines represent relationships within each habitat type. Black solid lines indicate statistically significant relationships derived from linear mixed-effects models with random intercepts, with shaded areas representing 95% confidence intervals.


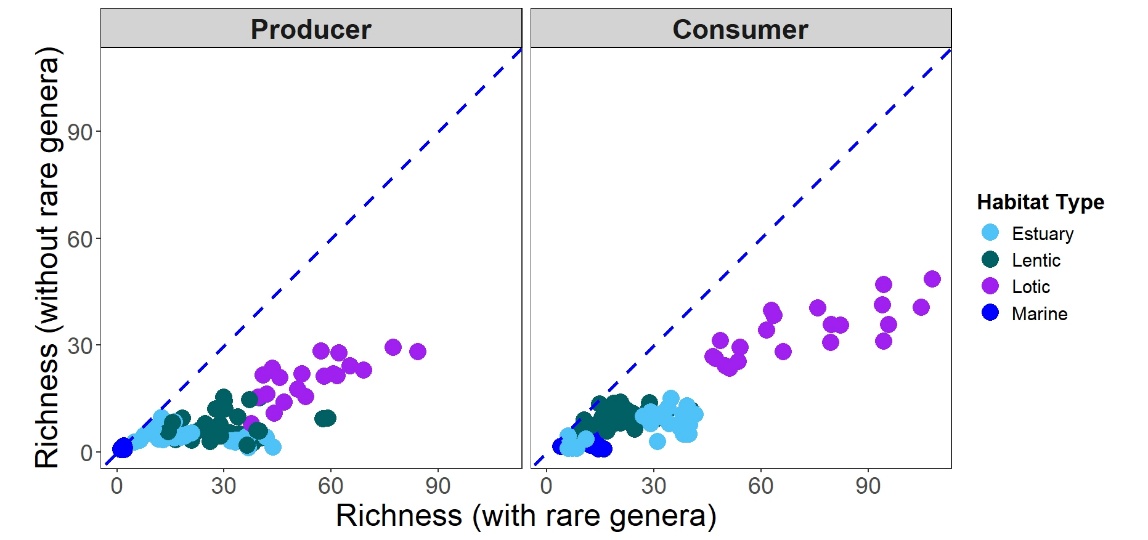


**Fig. S7**. Relationship between taxonomic richness without vs. with extremely rare genera (i.e., those with average population size < 1‰) in producer (a) and consumer (b) communities.

**
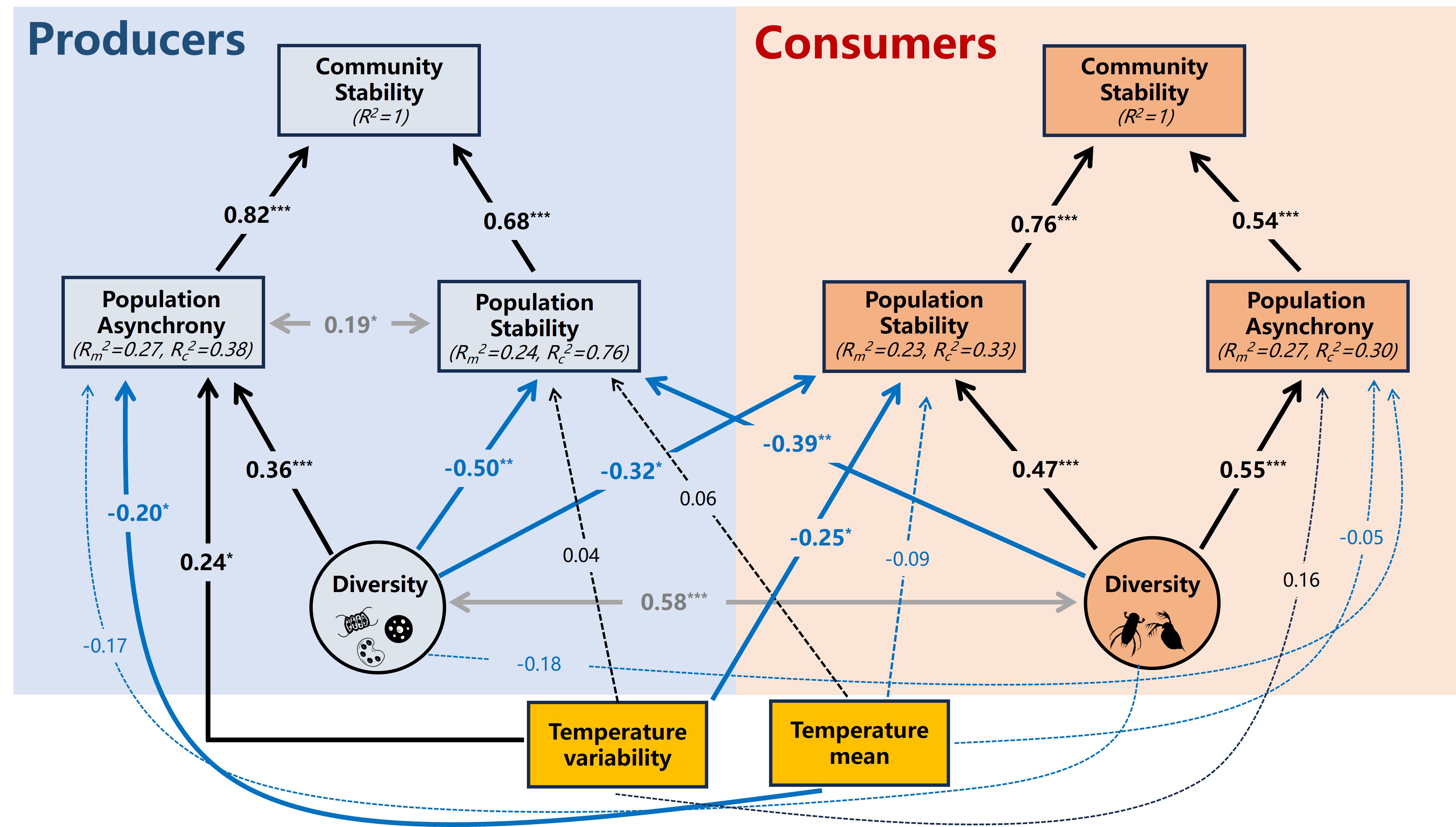
**

**Fig. S8 Full structural equation model (SEM) illustrating the relationships among Simpson diversity, stability metrics and temperature variation across two trophic levels**. Positive associations are indicated by black arrows, while negative associations are shown with blue arrows. Gray arrows represent bivariate correlations. The solid lines suggested significant pathways (P<0.05) and their coefficients, while the dashed lines refer to non-significant pathways and their coefficients. The proportions of variance explained by the linear mixed models are denoted by marginal R² (R_m_²), which represents the variance explained by the fixed effects only, and conditional R² (R_c_²), which accounts for both fixed and random effects. All stability indices are ln-transformed prior to analysis. The final model demonstrated good fit with Fisher's C = 24.88, degrees of freedom (d.f.) = 32, and P = 0.81.

**Tables**

**Table S1** Information of datasets included in the present study.

| **Site name** | **Year length** | **Latitude** | **Longitude** | **Country** | **Site type** | **Source** |
| --- | --- | --- | --- | --- | --- | --- |
| Nors Sø | 19 | 57.03044 | 8.609856 | Denmark | Lentic  Freshwaters | Provided by Erik Jeppesen |
| Hornum Sø | 19 | 56.90216 | 9.748081 | Denmark | Lentic  Freshwaters | Provided by Erik Jeppesen |
| Hinge Sø | 20 | 56.25676 | 9.500554 | Denmark | Lentic  Freshwaters | Provided by Erik Jeppesen |
| Ravnsø | 19 | 56.10573 | 9.845287 | Denmark | Lentic  Freshwaters | Provided by Erik Jeppesen |
| Bryrup Langsø | 19 | 56.01605 | 9.530448 | Denmark | Lentic  Freshwaters | Provided by Erik Jeppesen |
| Søby Sø | 20 | 56.05131 | 9.072553 | Denmark | Lentic  Freshwaters | Provided by Erik Jeppesen |
| Holm Sø | 19 | 55.64534 | 8.207144 | Denmark | Lentic  Freshwaters | Provided by Erik Jeppesen |
| Kvie Sø | 19 | 55.72406 | 8.769453 | Denmark | Lentic  Freshwaters | Provided by Erik Jeppesen |
| Engelsholm Sø | 19 | 55.72207 | 9.318557 | Denmark | Lentic  Freshwaters | Provided by Erik Jeppesen |
| Dons Sø | 16 | 55.55648000 | 9.42669 | Denmark | Lentic  Freshwaters | Provided by Erik Jeppesen |
| Søgørd Sø | 19 | 55.42932 | 9.321553 | Denmark | Lentic  Freshwaters | Provided by Erik Jeppesen |
| Store Søgørd Sø | 19 | 54.93897 | 9.447043 | Denmark | Lentic  Freshwaters | Provided by Erik Jeppesen |
| Arreskov Sø | 20 | 55.16120 | 10.311241 | Denmark | Lentic  Freshwaters | Provided by Erik Jeppesen |
| Søholm Sø | 20 | 55.27396 | 10.131158 | Denmark | Lentic  Freshwaters | Provided by Erik Jeppesen |
| Arresø | 19 | 55.97648 | 12.122505 | Denmark | Lentic  Freshwaters | Provided by Erik Jeppesen |
| Furesø | 19 | 55.80399 | 12.421367 | Denmark | Lentic  Freshwaters | Provided by Erik Jeppesen |
| Maglesø | 18 | 55.63235 | 11.690762 | Denmark | Lentic  Freshwaters | Provided by Erik Jeppesen |
| Gundsømagle Sø | 19 | 55.72750 | 12.190874 | Denmark | Lentic  Freshwaters | Provided by Erik Jeppesen |
| Utterslev Mose | 18 | 55.71573 | 12.499284 | Denmark | Lentic  Freshwaters | Provided by Erik Jeppesen |
| Tissø | 19 | 55.57170 | 11.290557 | Denmark | Lentic  Freshwaters | Provided by Erik Jeppesen |
| Vesterborg Sø | 20 | 54.86496 | 11.273756 | Denmark | Lentic  Freshwaters | Provided by Erik Jeppesen |
| Santa Barbara Coastal site 1 | 20 | 34.40028 | -119.744591 | U.S. | Marine | 14 |
| Santa Barbara Coastal site 2 | 20 | 34.47182 | -120.142617 | U.S. | Marine | 14 |
| Santa Barbara Coastal site 3 | 19 | 34.46775 | -120.119050 | U.S. | Marine | 14 |
| Arikaree River | 6 | 39.75821 | -102.447148 | U.S. | Lotic freshwaters | 15-17 |
| Lake Barco | 4 | 29.67607 | -82.008175 | U.S. | Lentic  Freshwaters | 15-17 |
| Blacktail Deer Creek | 3 | 44.95011 | -110.587150 | U.S. | Lotic freshwaters | 15-17 |
| Blue River | 3 | 34.44422 | -96.624201 | U.S. | Lotic freshwaters | 15-17 |
| Santa Barbara Coastal site 4 | 21 | 34.45851 | -120.333490 | U.S. | Marine | 14 |
| Bush River | 19 | 39.36090 | -76.253700 | U.S. | Estuary | 18-20; provided by Qinghua Zhao |
| Caribou Creek | 4 | 65.15322 | -147.503973 | U.S. | Lotic freshwaters | 15-17 |
| Santa Barbara Coastal site 5 | 21 | 34.39163 | -119.541693 | U.S. | Marine | 14 |
| Como Creek | 5 | 40.03496 | -105.544164 | U.S. | Lotic freshwaters | 15-17 |
| Crystal Lake | 12 | 46.00292 | -89.610069 | U.S. | Lentic freshwaters | 21-22 |
| Río Cupeyes | 5 | 18.11352 | -66.986760 | U.S. | Lotic freshwaters | 15-17 |
| Choptank River | 19 | 38.58070 | -76.058700 | U.S. | Estuary | 18-20; provided by Qinghua Zhao |
| Sacramento-San Joaquin Delta site 10 | 14 | 38.08257 | -121.570798 | U.S. | Estuary | 23-24 |
| Sacramento-San Joaquin Delta site 11 | 14 | 37.97663 | -121.575111 | U.S. | Estuary | 23-24 |
| Sacramento-San Joaquin Delta site 12 | 14 | 38.06206 | -121.821216 | U.S. | Estuary | 23-24 |
| Sacramento-San Joaquin Delta site 15 | 14 | 38.04835 | -122.112098 | U.S. | Estuary | 23-24 |
| Sacramento-San Joaquin Delta site 16 | 14 | 38.10844 | -122.045789 | U.S. | Estuary | 23-24 |
| Sacramento-San Joaquin Delta site 17 | 14 | 38.06291 | -121.988907 | U.S. | Estuary | 23-24 |
| Sacramento-San Joaquin Delta site 18 | 14 | 38.04294 | -121.423144 | U.S. | Estuary | 23-24 |
| Sacramento-San Joaquin Delta site 19 | 3 | 38.06278 | -122.211072 | U.S. | Estuary | 23-24 |
| Sacramento-San Joaquin Delta site 20 | 5 | 38.03512 | -122.152494 | U.S. | Estuary | 23-24 |
| Sacramento-San Joaquin Delta site 21 | 5 | 38.17424 | -122.016990 | U.S. | Estuary | 23-24 |
| Sacramento-San Joaquin Delta site 22 | 5 | 38.13751 | -121.691597 | U.S. | Estuary | 23-24 |
| Sacramento-San Joaquin Delta site 23 | 6 | 38.05647 | -122.290843 | U.S. | Estuary | 23-24 |
| Sacramento-San Joaquin Delta site 24 | 5 | 38.17728 | -122.049726 | U.S. | Estuary | 23-24 |
| Sacramento-San Joaquin Delta site 25 | 14 | 37.97294 | -121.376130 | U.S. | Estuary | 23-24 |
| Sacramento-San Joaquin Delta site 4 | 5 | 38.04667 | -121.920498 | U.S. | Estuary | 23-24 |
| Sacramento-San Joaquin Delta site 5 | 6 | 38.02455 | -121.816095 | U.S. | Estuary | 23-24 |
| Sacramento-San Joaquin Delta site 6 | 6 | 38.09867 | -121.682444 | U.S. | Estuary | 23-24 |
| Sacramento-San Joaquin Delta site 7 | 13 | 38.04463 | -121.615637 | U.S. | Estuary | 23-24 |
| Sacramento-San Joaquin Delta site 8 | 6 | 38.08640 | -121.736731 | U.S. | Estuary | 23-24 |
| East Long Lake | 6 | 46.23640 | -89.494800 | U.S. | Lentic freshwaters | 25-26 |
| Elk River | 19 | 39.44270 | -76.000600 | U.S. | Estuary | 18-20; provided by Qinghua Zhao |
| Fuxian Lake | 4 | 24.50218 | 102.889750 | China | Lentic freshwaters | Upon request |
| Santa Barbara Coastal site 6 | 20 | 34.41372 | -119.822100 | U.S. | Marine | 14 |
| Río Yahuecas | 5 | 18.17406 | -66.798680 | U.S. | Lotic freshwaters | 15-17 |
| Harvard Forest & Quabbin Watershed | 4 | 42.47194 | -72.329526 | U.S. | Lotic freshwaters | 15-17 |
| Santa Barbara Coastal site 7 | 10 | 34.40278 | -119.857550 | U.S. | Marine | 14 |
| Kings Creek | 6 | 39.10506 | -96.603829 | U.S. | Lotic freshwaters | 15-17 |
| LeConte Creek | 5 | 35.69043 | -83.503790 | U.S. | Lotic freshwaters | 15-17 |
| Lewis Run | 5 | 39.09564 | -77.983216 | U.S. | Lotic freshwaters | 15-17 |
| TaiHu Lake | 11 | 31.33259 | 120.164177 | China | Lentic freshwaters | Upon request |
| Mayfield Creek | 5 | 32.96037 | -87.407688 | U.S. | Lotic freshwaters | 15-17 |
| McDiffett Creek | 4 | 38.94586 | -96.443022 | U.S. | Lotic freshwaters | 15-17 |
| McRae Creek | 3 | 44.25960 | -122.165554 | U.S. | Lotic freshwaters | 15-17 |
| Lake Mendota | 24 | 43.14600 | -89.367339 | U.S. | Lentic freshwaters | 27-28 |
| Lake Monona | 23 | 43.07117 | -89.357360 | U.S. | Lentic freshwaters | 27-28 |
| Santa Barbara Coastal site 8 | 20 | 34.39407 | -119.729570 | U.S. | Marine | 14 |
| Magothy River | 19 | 39.04800 | -76.407000 | U.S. | Estuary | 18-20; provided by Qinghua Zhao |
| Santa Barbara Coastal site 9 | 21 | 34.42212 | -119.951540 | U.S. | Marine | 14 |
| Oksrukuyik Creek | 5 | 68.66975 | -149.143018 | U.S. | Lotic freshwaters | 15-17 |
| Posey Creek | 7 | 38.89431 | -78.147258 | U.S. | Lotic freshwaters | 15-17 |
| Pringle Creek | 4 | 33.37852 | -97.782312 | U.S. | Lotic freshwaters | 15-17 |
| Dakota Coteau Field Site | 3 | 47.15989 | -99.117722 | U.S. | Lentic freshwaters | 15-17 |
| Prairie Pothole | 3 | 47.13049 | -99.252995 | U.S. | Lentic freshwaters | 15-17 |
| Patapsco River | 19 | 39.21300 | -76.522500 | U.S. | Estuary | 18-20; provided by Qinghua Zhao |
| Patuxent River | 19 | 38.31270 | -76.455000 | U.S. | Estuary | 18-20; provided by Qinghua Zhao |
| Paul Lake | 15 | 46.25145 | -89.503468 | U.S. | Lentic freshwaters | 25-26 |
| Peter Lake | 15 | 46.25320 | -89.503500 | U.S. | Lentic freshwaters | 25-26 |
| Potomac River | 19 | 38.15760 | -76.598000 | U.S. | Estuary | 18-20; provided by Qinghua Zhao |
| Red Butte Creek | 5 | 40.78393 | -111.797887 | U.S. | Lotic freshwaters | 15-17 |
| Santa Barbara Coastal site 10 | 17 | 34.05865 | -119.757630 | U.S. | Marine | 14 |
| Santa Barbara Coastal site 11 | 17 | 34.04443 | -119.715130 | U.S. | Marine | 14 |
| Sparkling Lake | 12 | 46.09100 | -89.699200 | U.S. | Lentic freshwaters | 21-22 |
| Lake Suggs | 4 | 29.68695 | -82.018776 | U.S. | Lentic freshwaters | 15-17 |
| Sycamore Creek | 3 | 33.75099 | -111.508091 | U.S. | Lotic freshwaters | 15-17 |
| South River | 19 | 38.90430 | -76.480500 | U.S. | Estuary | 18-20; provided by Qinghua Zhao |
| Tuesday Lake | 10 | 46.25120 | -89.497200 | U.S. | Lentic freshwaters | 25-26 |
| Walker Branch | 5 | 35.95738 | -84.279251 | U.S. | Lotic freshwaters | 15-17 |
| West St Louis Creek | 3 | 39.89137 | -105.915395 | U.S. | Lotic freshwaters | 15-17 |
| West Long Lake | 6 | 46.23600 | -89.502100 | U.S. | Lentic freshwaters | 25-26 |
| York river | 19 | 37.23450 | -76.423400 | U.S. | Estuary | 18-20; provided by Qinghua Zhao |
| Zurich Lake | 32 | 47.33000 | 8.560000 | Switzerland | Lentic freshwaters | 29 |

**Table S2** Effects of producer diversity, consumer diversity, annual mean and variation (standard deviation, SD) of temperature on community stability of both producer and consumer, derived from linear mixed models (LMERs).

| **Variable** | **Estimate** | **Stand Error** | **De-nDF** | **t value** | **Pr(>\|t\|)** |
| --- | --- | --- | --- | --- | --- |
| **Producer community stability as responding variable** | | | | | |
| Intercept | 1.028 | 0.526 | 17.48 | 1.954 | 0.0669 |
| Producer diversity | -0.374 | 0.359 | 70.5 | -1.043 | 0.300 |
| Consumer diversity | -1.272 | 0.463 | 70.0 | -2.746 | **0.008** |
| Annual temperature mean | -0.013 | 0.016 | 91.2 | -0.797 | 0.427 |
| Annual temperature variation | -0.112 | 0.063 | 89.9 | -1.768 | 0.080 |
| **Consumer community stability as responding variable** | | | | | |
| Intercept | -0.314 | 0.299 | 11.5 | -1.051 | 0.315 |
| Producer diversity | -0.677 | 0.255 | 53.5 | -2.658 | **0.010** |
| Consumer diversity | 1.923 | 0.317 | 32.8 | 6.06 | **<0.001** |
| Annual temperature mean | -0.008 | 0.013 | 26.6 | -0.598 | 0.555 |
| Annual temperature variation | -0.046 | 0.053 | 71.4 | -0.866 | 0.389 |

**Table S3** Effects of random effects, i.e., habitat type, on consumer and producer community stability based on linear mixed models (LMERs) results (Number of observations: 97, groups: habitat type, 4).

| **Groups** | **Variance** | **Stand deviation** |
| --- | --- | --- |
| **Producer community stability as responding variable** | | |
| Habitat type | 0.233 | 0.483 |
| Residual | 0.244 | 0.494 |
| **Consumer community stability as responding variable** | | |
| Habitat type | 0.001 | 0.038 |
| Residual | 0.197 | 0.443 |

**Table S4** Results from linear mixed models with random intercepts examining the effects of producer diversity, consumer diversity, annual mean temperature, and temperature variation (standard deviation, SD) on community stability of producers and consumers. Diversity was quantified using three metrics: Simpson index, Shannon index, and species richness. Analyses were conducted using two time windows: minimum 3-year and minimum 5-year periods. Rare genera were defined as those with relative abundance less than 1‰. Values presented are coefficients with significance levels: ***P<0.001, **P<0.01, *P<0.05, **.**P<0.1.

| **Diversity index** | **Responding variable** | **Producer diversity** | **Consumer diversity** | **Annual temperature mean** | **Annual temperature variation** |
| --- | --- | --- | --- | --- | --- |
| **Data with minimum sampling years of 3 years** | | | | | |
| **Simpson index** | Producer stability | -0.374 | **-1.272**** | -0.013 | -0.112**.** |
|  | Consumer stability | **-0.677*** | **1.923**** | -0.008 | -0.046 |
| **Shannon index** | Producer stability | -0.164 | **-0.393**** | 0.016 | 0.110 |
|  | Consumer stability | -0.164**.** | **0.435***** | -0.112 | -0.097**.** |
| **Richness without rare** **genera (ln- transformed)** | Producer stability | **0.411***** | **-0.218**** | -0.002 | 0.020 |
|  | Consumer stability | **-0.231**** | **0.469***** | -0.010 | -0.084**.** |
| **Richness (ln- transformed)** | Producer stability | **-0.253*** | -0.116 | -0.014 | 0.093 |
|  | Consumer stability | 0.188**.** | **0.503***** | -0.003 | -0.085**.** |
| **Data with minimum sampling years of 5 years** | | | | | |
| **Simpson index** | Producer stability | 0.103 | **-1.378**** | -0.011 | -0.010 |
|  | Consumer stability | **-0.602*** | **1.837***** | -0.006 | -0.105 |
| **Shannon index** | Producer stability | -0.062 | **-0.456**** | 0.013 | 0.018 |
|  | Consumer stability | -0.165 | **0.456***** | 0.215 | **0.989*** |
| **Richness without rare genera (ln-transformed)** | Producer stability | **0.534***** | **-0.300***** | 0.005 | **-0.131*** |
|  | Consumer stability | **-0.265**** | **0.493***** | -0.010 | 0.120**.** |
| **Richness (ln-transformed)** | Producer stability | **-0.804*** | **-0.245*** | -0.014 | -0.044 |
|  | Consumer stability | 0.005 | **0.496***** | -0.006 | **0.157*** |

**Table S5** Effects of habitat type, producer diversity, consumer diversity, and their interactions, as well as annual mean temperature and temperature variation (standard deviation, SD) on producer and consumer community stability based on multiple linear regression analysis with Type III ANOVA. Sum Sq = Sum of Squares; DF = Degrees of Freedom.

| **Variable** | **Sum Sq** | **DF** | **F value** | **Pr(>F)** |
| --- | --- | --- | --- | --- |
| **Producer community stability as responding variables** | | | | |
| Intercept | 3.119 | 1 | 13.412 | **<0.001** |
| Habitat type | 2.396 | 3 | 3.435 | 0.021 |
| Producer diversity | 1.778 | 1 | 7.643 | **0.007** |
| Consumer diversity | 2.753 | 1 | 11.836 | **<0.001** |
| Annual temperature mean | 0.250 | 1 | 1.076 | 0.303 |
| Annual temperature variation | 0.774 | 1 | 3.327 | 0.072 |
| Habitat type : producer Simpson | 1.572 | 3 | 2.253 | 0.088 |
| Habitat type : consumer Simpson | 0.736 | 3 | 1.054 | 0.373 |
| Residuals | 19.302 | 83 |  |  |
| **Consumer community stability as responding variables** | | | | |
| Intercept | 0.022 | 1 | 0.113 | 0.737 |
| Habitat type | 0.164 | 3 | 0.286 | 0.835 |
| Producer diversity | 1.986 | 1 | 10.410 | **0.002** |
| Consumer diversity | 2.080 | 1 | 10.908 | **0.001** |
| Annual temperature mean | 0.219 | 1 | 1.146 | 0.287 |
| Annual temperature variation | 0.136 | 1 | 0.713 | 0.401 |
| Habitat type : producer Simpson | 1.224 | 3 | 2.175 | 0.097 |
| Habitat type : consumer Simpson | 0.325 | 3 | 0.567 | 0.638 |
| Residuals | 15.831 | 83 |  |  |

**Table S6** Effects of producer diversity, consumer diversity, annual mean and variation (standard deviation, SD) of temperature on community stability of producer and consumer for data with different season numbers. Results were derived from linear mixed models (LMERs) with random intercept. Values shown are coefficients with significance levels indicated as: *P<0.05, **P<0.01, ***P<0.001.

| **Site selection** | Responding variable | Producer diversity | Consumer diversity | Annual temperature mean | Annual temperature variation |
| --- | --- | --- | --- | --- | --- |
| Sites surveyed over 4 seasons (n=60) | Producer stability | -0.488 | **-2.136**** | 0.009 | 0.115 |
|  | Consumer stability | **-0.888*** | **2.055***** | -0.004 | -0.070 |
| Sites surveyed over ≥ 3 seasons (n = 83) | Producer stability | -0.593 | **-1.867**** | -0.020 | 0.088 |
|  | Consumer stability | **-0.932**** | **2.083***** | -0.005 | -0.032***** |
| Sites surveyed over ≥ 2 seasons (n = 90) | Producer stability | -0.333 | **-1.490**** | -0.014 | 0.103 |
|  | Consumer stability | **-0.907***** | **2.012***** | -0.005 | -0.030***** |

**Table S7** Diversity-stability relationships after accounting for spatial autocorrelation. Results were derived from generalized additive mixed models (GAMMs) incorporating thin plate spline smooth functions for spatial coordinates (latitude and longitude) and random intercepts for habitat type.

| **Variable** | **Estimate** | **Stand Error** | **t value** | **Pr(>\|t\|)** |
| --- | --- | --- | --- | --- |
| **Producer community stability as responding variables** | | | | |
| Intercept | 2.027 | 0.572 | 3.54 | **0.0006** |
| Producer diversity | -0.523 | 0.342 | -1.53 | 0.130 |
| Consumer diversity | -1.214 | 0.437 | -2.778 | **0.007** |
| Annual temperature mean | -0.082 | 0.025 | -3.22 | **0.002** |
| Annual temperature variation | 0.133 | 0.060 | 2.21 | **0.030** |
| **Consumer community stability as responding variables** | | | | |
| Intercept | -0.030 | 0.394 | 0.075 | 0.940 |
| Producer diversity | -0.637 | 0.251 | -2.54 | **0.013** |
| Consumer diversity | 1.987 | 0.310 | 6.42 | **<0.001** |
| Annual temperature mean | -0.035 | 0.024 | -1.48 | 0.142 |
| Annual temperature variation | -0.050 | 0.052 | -0.952 | 0.344 |

**References**

1. Harris IC, Jones PD, Osborn T. CRU TS4.07: Climatic Research Unit (CRU) Time-Series (TS) version 4.07 of high-resolution gridded data of month-by-month variation in climate (Jan. 1901- Dec. 2022). NERC EDS Centre for Environmental Data Analysis 2023.

2. Huang B, Thorne PW, Banzon VF et al. NOAA Extended Reconstructed Sea Surface Temperature (ERSST), Version 5. NOAA National Centers for Environmental Information 2017; 30: 8179–8205.

3. Piccolroaz S, Toffolon M, Majone B. A simple lumped model to convert air temperature into surface water temperature in lakes. Hydrol Earth Syst Sci 2013; 17: 3323–3338.

4. Tilman D, Reich PB, Knops JMH. Biodiversity and ecosystem stability in a decade-long grassland experiment. Nature 2006; 441: 629-632.

5. Thibaut LM, Connolly SR. Understanding diversity–stability relationships: towards a unified model of portfolio effects. Ecol Lett 2013; 16: 140–150.

6. Arnoldi J, Loreau M, Haegeman B. The inherent multidimensionality of temporal variability: how common and rare species shape stability patterns. Ecol Lett 2019; 22: 1557–1567.

7. Wang S, Loreau M. Biodiversity and ecosystem stability across scales in metacommunities. Ecol Lett 2016; 19: 510–518.

8. Jaeger BC, Edwards LJ, Das K et al. An R² statistic for fixed effects in the generalized linear mixed model. J Appl Stat 2017; 44: 1086–1105.

9. Bates D, Mächler M, Bolker B et al. Fitting Linear Mixed-Effects Models Using lme4. J Stat Softw 2015; 67: 1–48.

10. Lefcheck JS. piecewiseSEM: Piecewise structural equation modelling in r for ecology, evolution, and systematics. Methods Ecol Evol 2016; 7: 573–579.

11. Zhao J, Brandt G, Gronniger JL et al. Quantifying the contribution of the rare biosphere to natural disturbances. ISME J 2025; 19: wraf129.

12. Cabrera M, Taylor G. Modelling spatio-temporal data of dengue fever using generalized additive mixed models. Spat Spatio-temporal Epidemiol 2019; 28: 1–13.

13. R Core Team. R: A Language and Environment for Statistical Computing (R Foundation for Statistical Computing, 2019).

1. Santa Barbara Coastal LTER, D. Reed, and R. Miller. 2021. SBC LTER: Reef: Annual time series of biomass for kelp forest species, ongoing since 2000 (Reformatted to the ecocomDP Design Pattern) ver 3. Environmental Data Initiative. https://doi.org/10.6073/pasta/4392212f4d40253672acc47d4b3b9c13 (Accessed 2025-01-16).
2. NEON (National Ecological Observatory Network). Periphyton, seston, and phytoplankton chemical properties (DP1.20163.001), RELEASE-2024. https://doi.org/10.48443/25wy-9f31. Dataset accessed from https://data.neonscience.org/data-products/DP1.20163.001/RELEASE-2024 on January 16, 2025.
3. NEON (National Ecological Observatory Network). Zooplankton collection (DP1.20219.001), RELEASE-2024. https://doi.org/10.48443/6kxg-hb11. Dataset accessed from https://data.neonscience.org/data-products/DP1.20219.001/RELEASE-2024 on January 16, 2025.
4. NEON (National Ecological Observatory Network). Macroinvertebrate metabarcoding (DP1.20126.001), RELEASE-2024. https://doi.org/10.48443/gkx2-x153. Dataset accessed from https://data.neonscience.org/data-products/DP1.20126.001/RELEASE-2024 on January 16, 2025.
5. Harding LW et al. Long-Term Trends of Nutrients and Phytoplankton in Chesapeake Bay. Estuar Coasts 2016; 39: 664–681.
6. Slater WL, Pierson JJ, Decker MB et al. Fewer Copepods, Fewer Anchovies, and More Jellyfish: How Does Hypoxia Impact the Chesapeake Bay Zooplankton Community? Diversity 2020; 12: 35.
7. Marshall HG, Burchardt L, Lacouture R. A review of phytoplankton composition within Chesapeake Bay and its tidal estuaries. J Plankton Res 2005; 27: 1083–1102.
8. Magnuson J, Carpenter SR, Stanley E. 2023. North Temperate Lakes LTER: Phytoplankton - Trout Lake Area 1984 - 2006 ver 20. Environmental Data Initiative. https://doi.org/10.6073/pasta/e876543ebe841bf7608f800a6cfb357e (Accessed 2025-01-16).
9. Magnuson JJ, Carpenter SR, Stanley EH. 2023. North Temperate Lakes LTER: Zooplankton - Trout Lake Area 1982 - current ver 38. Environmental Data Initiative. https://doi.org/10.6073/pasta/04030c61782378dd44abb005b29ea5d3 (Accessed 2025-01-16).
10. Bashevkin SM, Hartman R, Thomas M et al. 2023. Interagency Ecological Program: Zooplankton abundance in the Upper San Francisco Estuary from 1972-2021, an integration of 7 long-term monitoring programs ver 4. Environmental Data Initiative. https://doi.org/10.6073/pasta/8b646dfbeb625e308212a39f1e46f69b (Accessed 2025-01-16).
11. Perry SE, Brown T, Klotz V. 2024. Interagency Ecological Program: Phytoplankton monitoring in the Sacramento-San Joaquin Bay-Delta, collected by the Environmental Monitoring Program, 2008-2023 ver 9. Environmental Data Initiative. https://doi.org/10.6073/pasta/3f08391bdf51583bd4282e697485201d (Accessed 2025-01-16).
12. Carpenter SR, Kitchell J, Cole J et al. 2024. Cascade Project at North Temperate Lakes LTER Core Data Zooplankton 1984 - 2019 ver 6. Environmental Data Initiative. https://doi.org/10.6073/pasta/800a885203365c03f90d2fff58376862 (Accessed 2025-01-16).
13. Carpenter SR, Kitchell J, Cole J et al. 2022. Cascade Project at North Temperate Lakes LTER Core Data Phytoplankton 1984 - 2015 ver 5. Environmental Data Initiative. https://doi.org/10.6073/pasta/05074f1e58c54e2f71b0b3774e9a90bc (Accessed 2025-01-16).
14. Magnuson JJ, Carpenter SR, Stanley EH. 2022. North Temperate Lakes LTER: Phytoplankton-Madison Lakes Area 1995 - current ver 31. Environmental Data Initiative. https://doi.org/10.6073/pasta/bd03330e83fc52a5442bb39e131c95e9 (Accessed 2025-01-16).
15. Magnuson J, Carpenter SR, Stanley E. 2024. North Temperate Lakes LTER: Zooplankton Madison Lakes Area 1997 - current ver 36. Environmental Data Initiative. https://doi.org/10.6073/pasta/a03b42039e6db8c45a9bb9e480a2ba6a (Accessed 2025-01-16).
16. Dornelas M et al. BioTIME: A database of biodiversity time series for the Anthropocene. Glob Ecol Biogeogra 2018; 27:760–786.
